# Colorectal mucosal exposure area assessment using artificial intelligence: a multicenter prospective observational study

**DOI:** 10.1055/a-2695-1832

**Published:** 2025-10-17

**Authors:** Jialing Li, Li Huang, Chaijie Luo, Xiaoquan Zeng, Ying Li, Jianping Fan, Liwen Yao, Jing Wang, Shuzhe Tan, Xueying Wang, Wei Zhou, Lianlian Wu, Dexin Gong, Yuliang Xu, Muqiu Li, Ningning Wang, Huafen Gao, Honggang Yu

**Affiliations:** 1 117921 Department of Gastroenterology, Renmin Hospital of Wuhan University, Wuhan, China; 2 117921 Key Laboratory of Hubei Province for Digestive System Disease, Renmin Hospital of Wuhan University, Wuhan, China; 3 117921 Hubei Provincial Clinical Research Center for Digestive Disease Minimally Invasive Incision, Renmin Hospital of Wuhan University, Wuhan, China; 4 Engineering Research Center for Artificial Intelligence Endoscopy Interventional Treatment of Hubei Province, Wuhan, China; 5 735636 Department of Endoscopy, The Eighth Hospital of Wuhan, Wuhan, China; 6 Endoscopy Center, JingXing County Hospital, Hebei, China; 7 117921 Department of Gastroenterology, Wuhan University Renmin Hospital, Wuhan, China; 8 117921 Key Laboratory of Hubei Province for Digestive System Disease, Wuhan University Renmin Hospital, Wuhan, China; 9 117921 Hubei Provincial Clinical Research Center for Digestive Disease Minimally Invasive Incision, Wuhan University Renmin Hospital, Wuhan, China; 10 71069 Department of Gastroenterology, Zhejiang University School of Medicine First Affiliated Hospital, Hangzhou, China

## Abstract

**Background:**

This study proposed a new quality control indicator for colonoscopy, the cumulative colorectal mucosal exposure area (CCMEA), to assess mucosal exposure, constructed a CCMEA system based on deep learning, and validated the indicator in a multicenter prospective observational study.

**Methods:**

The CCMEA system was based on ResNet50 and UNet++. A CCMEA threshold was determined on the basis of an adenoma detection rate (ADR) of 25%. A multicenter prospective observational study was conducted to evaluate the system and the threshold in clinical practice. Based on the CCMEA threshold, patients were divided into qualified and unqualified colonoscopy groups. The ADR and other lesion detection rates were then compared between the two groups.

**Results:**

510 participants who underwent colonoscopy were evaluated, being grouped as having qualified (n = 270) or unqualified (n = 240) colonoscopies based on a CCMEA qualification threshold of 2000. The ADR was 39.5 percentage points higher in the qualified group than in the unqualified group (53.7% vs. 14.2%; adjusted odds ratio [aOR] 8.0, 95%CI 5.0–12.8; *P* < 0.001), and notably was higher for lesions ≤5 mm (42.2% vs. 10.0%; aOR 6.9, 95%CI 4.1–11.5; *P* < 0.001). The qualified group also had a significantly higher polyp detection rate (89.6% vs. 40.0%; aOR 13.1, 95%CI 7.8–21.8; *P* < 0.001) and higher mean numbers of both adenomas (1.0 vs. 0.2; adjusted incident rate ratio [aIRR] 5.9, 95%CI 4.3–8.4; *P* < 0.001) and polyps (5.8 vs. 1.3; aIRR 4.0, 95%CI 3.5–4.5; *P* < 0.001).

**Conclusions:**

The CCMEA qualified group, based on a CCMEA threshold of 2000, showed a higher ADR than the unqualified group, indicating CCMEA could be a promising colonoscopy quality indicator.

## Abstract (de)

**Graphical Abstract:**

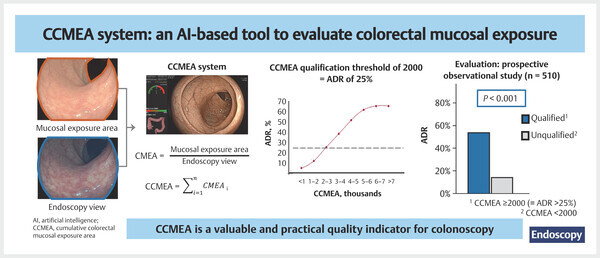

## Introduction

Colonoscopy is generally considered the gold standard method for colorectal cancer (CRC) screening, which can decrease the incidence and mortality of CRC by 40%–50% 1 2 . However, colonoscopy is still associated with a high miss rate of colorectal neoplasia, and the adenoma detection rate (ADR) varies among operators. It is increasingly recognized that the effectiveness of the procedure relies on the quality of the colonoscopy. Most current guidelines for surveillance intervals are based on high quality colonoscopy; suboptimal quality is associated with a higher risk of missed adenomas and serrated lesions, which are linked to postcolonoscopy CRC (PCCRC) 3 .

Various quality indicators have been introduced for colonoscopy, including adequate withdrawal time, adequate bowel preparation, and ADR 4 . Traditional quality control indicators must be assessed by endoscopists to ensure clinical efficacy and increase accuracy. The introduction of artificial intelligence (AI) allows for the development of new AI-assisted quality control indicators that provide more effective evaluations 5 6 7 . For instance, effective withdrawal time has been proposed, using AI to automatically eliminate unqualified intervals during the withdrawal phase, thereby enhancing the efficacy of quality indicators 7 . Manual calculation of the effective withdrawal time is challenging, but AI can do this task swiftly and conveniently, offering a stronger correlation with ADR than the traditional indicator of standard withdrawal time.

To our knowledge, there are no studies on the accurate and automated assessment of colorectal mucosal exposure. Based on an AI system, we propose a novel colonoscopy quality indicator, namely the cumulative colorectal mucosal exposure area (CCMEA), to evaluate mucosal exposure during the withdrawal observation period. We designed a prospective observational study in which participants were divided into two groups based on the identified CCMEA threshold, with comparisons made of the differences between the two groups.

## Methods

### Establishment and threshold determination of the CCMEA system

**Fig. 1:**
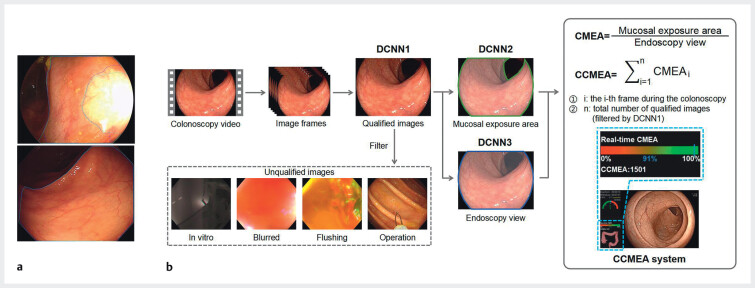
Images demonstrating the development of the cumulative colorectal mucosal exposure area (CCMEA) system showing: **a** example segmentation of the colorectal mucosal exposure area (the cyan line is the area of colorectal mucosal exposure labeled by the endoscopist; the blue line is the model prediction); **b** the stages of the CCMEA system, with example qualified and unqualified images. DCNN, deep convolutional neural network.

The colorectal mucosal exposure area (CMEA) is defined as the proportion of clearly exposed mucosa within a single colonoscopy frame relative to the entire field of view. This definition ensures that any polyp in the field can be immediately recognized by an endoscopist. Regions obscured by reflection, fecal material, occlusion, or excessive darkness will not be annotated as well-exposed mucosa. Fig. 1 **a** shows the representative CMEA in single image frames. The CMEA was calculated as follows: CMEA = mucosal exposure area/endoscopy view. The cumulative measure, the CCMEA, was defined as the sum of the CMEAs of all qualified colonoscopy frames during the complete withdrawal process.

The CCMEA system consists of three deep convolutional neural network (DCNN) models ( Fig. 1 **b** ). DCNN1 was employed to filter out unqualified images, including in vitro, blurred, flushing, instrument, operation, or chromoendoscopy images. Its performance was comprehensively validated in our previous study, where ResNet50 was employed as the backbone network, achieving an accuracy of 98.9% 5 . DCNN2 segmented the clearly exposed mucosa in the endoscopy view of qualified images, which were used as the molecules for calculation of the CMEA. During routine endoscopic procedures, endoscopists conducted the withdrawal procedure using white-light endoscopy, while chromoendoscopy was mainly used for diagnosis. Therefore, CCMEA calculations were discontinued when chromoendoscopy was in use. DCNN3 was used to segment the endoscopy view of the qualified image within the frame, which was used as the denominator of CMEA calculation to rule out the bias caused by the inconsistency of image regions between different endoscope manufacturers or types.

For DCNN2 and DCNN3, we used 3597 and 104 colonoscopy images, respectively, from the Endoscopy Center of Renmin Hospital of Wuhan University (RHWU) for image labeling and model training. Four endoscopists with more than 3 years of colonoscopy experience used an online image annotation tool, VGG Image Annotator ( https://www.robots.ox.ac.uk/~vgg/software/via/via-2.0.11.html ), for labeling. The final review was conducted by a senior endoscopist (≥10 years’ experience). Both the DCNN2 and DCNN3 adopted UNet++ as their framework architecture (details are available in **Appendix 1s** , see online-only Supplementary material).

**Fig. 2:**
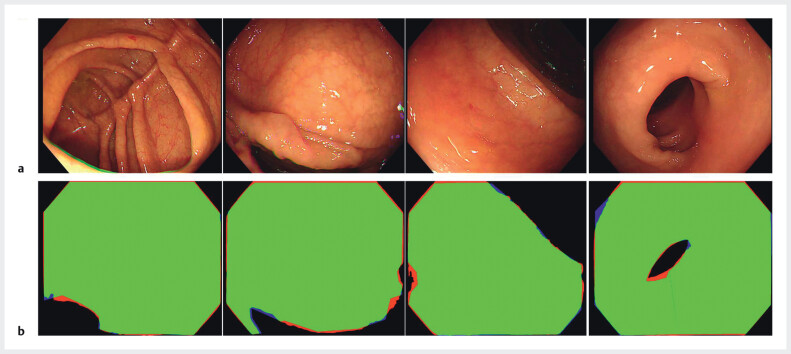
Visualization of the colorectal mucosal exposure area segmentation showing: **a** the original endoscopic images obtained by colonoscopy; **b** comparative results of area labeling, where the green area indicates the overlapping area for endoscopist labeling and system prediction, the blue areas represent the mucosal exposure range manually labeled by the endoscopist only, and the red areas represent the mucosal region automatically predicted by the cumulative colorectal mucosal exposure area (CCMEA) system only.

The Dice coefficient and mean Intersection over Union (mIoU) were adopted to assess the performance of the segmentation models. DCNN2 had a Dice coefficient of 0.93 and mIoU of 0.87; DCNN3 had a Dice coefficient of 0.99 and mIoU of >0.99. Fig. 2 shows the segmentation of the CMEA by DCNN2 in a single colonoscopy image.

We retrospectively collected colonoscopy videos from 716 patients who underwent colonoscopy at the Endoscopy Center of RHWU between March 2024 and August 2024 to validate the system performance. We employed this dataset to explore the CCMEA threshold for qualified colonoscopy. The eligibility criteria were patients aged >18 years with complete colonoscopy videos available. Patients who did not undergo biopsy, were pregnant or breastfeeding, had polyp syndromes, inflammatory bowel disease (IBD), a history of colorectal surgery, CRC, or incomplete cecal intubation were excluded.

**Fig. 3:**
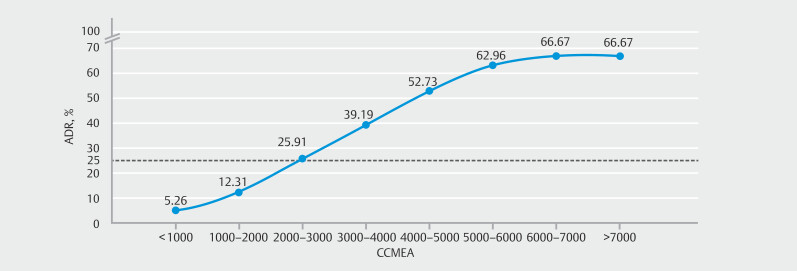
Threshold curve for the cumulative colorectal mucosal exposure area (CCMEA) grouped in increments of 1000. A CCMEA threshold of 2000 was chosen to guarantee an adenoma detection rate (ADR) of >25%.

For the purpose of this study, a per-colonoscopy definition was established. All colonoscopy videos in the threshold dataset were analyzed by the CCMEA system, and patients in the dataset were grouped by CCMEA in intervals of 1000. The qualified CCMEA threshold was determined based on the 25% ADR criterion for colonoscopy screening. As shown in Fig. 3 , which presents the fitted curve between CCMEA groupings and ADR, a CCMEA of 2000 was selected as the threshold to ensure that ADR exceeded 25%, thereby enabling higher quality endoscopic examinations. Within this study, a "qualified colonoscopy" therefore refers to an examination with a CCMEA value ≥2000, a threshold that corresponds to better visualization of the colorectal mucosa, as well as reliable adenoma detection. An "unqualified colonoscopy" refers to an examination with a CCMEA value <2000, where mucosal visualization may be insufficient to guarantee effective adenoma identification.

### Study design and participants

A prospective multicenter observational study was conducted to objectively evaluate the threshold of the CCMEA and assess the utility of the CCMEA system in clinical practice. Between October 2024 and November 2024, consecutive patients aged >18 years undergoing colonoscopy were recruited at three healthcare centers (RHWU, Wuhan Eighth Hospital [WEH], and JingXing County Hospital [JXCH]). The inclusion criteria were as follows: age ≥18 years; colonoscopy performed; ability to read, understand and sign the informed consent; the investigators believed that the participant could understand the process of this clinical study, and was willing and able to cooperate and complete all research processes. The exclusion criteria were as follows: pregnant or breastfeeding; drug or alcohol abuse or mental illness in the past 5 years; previous colorectal surgery; multiple polyp syndrome; IBD; bowel stricture; CRC; bowel obstruction or perforation; failure to reach the cecum during the procedure; contraindications to biopsy; polyposis syndrome or IBD having been suspected during the procedure.

### Procedure

Patients who met the inclusion and exclusion criteria and provided informed consent were enrolled in the study. The basic demographic characteristics of the participants, including sex, age, body mass index (BMI), and indication for colonoscopy, were recorded before colonoscopy.

Patients underwent routine colonoscopy procedures. BL-7000 (Fujifilm, Kanagawa, Japan) and CF-HQ290/CF-Q260AI (Olympus Optical, Tokyo, Japan) colonoscopes were used in this study. Biopsies were taken for histological examination after polyps were detected. The CCMEA system was employed to assess and record the CCMEA during the examination. The researchers documented the results as they occurred in real-time. Colonoscopy videos, endoscopy reports, and pathology reports were collected following the colonoscopy. The pathological diagnosis was regarded as the gold standard.

### Ethics statement

This study was approved by the Ethics Committee of RHWU (No. WDRY2024-K071). All participants signed the informed consent form.

### Outcome

The primary outcome of this study was the ADR, which was defined as the proportion of patients in whom at least one pathologically confirmed adenoma was detected during colonoscopy. Secondary outcomes were: the detection rates for adenomas of different sizes (diminutive, ≤5 mm; small, >5 to <10 mm; large, ≥10 mm), the polyp detection rate (PDR), the detection rates for polyps of different sizes, detection rates for advanced adenomas (lesion diameter ≥10 mm, high grade dysplasia, or villous histology), and mean numbers of adenomas or polyps per patient.

### Sample size

This study primarily aimed to investigate the relationship between CCMEA and ADR. There was no previous study regarding the distribution of CCMEA thresholds and ADR. Therefore, based on prior experience with quality control indicators and historical ADR data from the three participating healthcare centers (RHWU, WEH, and JXCH), we assumed an ADR of 33% in the qualified group and 23% in the unqualified group, with a 10 percentage point difference or greater between the two groups 5 6 8 9 10 .

PASS 15.0 (NCSS Statistical Software, Kaysville, Utah, USA) was employed to calculate the sample size. Based on a 10% dropout rate, 550 participants were determined as being necessary to achieve 74% power at a significance level of α = 0.05 for differences in ADR between the two groups.

#### Statistical analysis

The prospective datasets were divided into a qualified group (above the threshold) and an unqualified group (below the threshold) based on the determined CCMEA threshold for analysis. Continuous variables are presented as mean and SD and categorical variables as frequencies and percentages. For baseline comparisons, continuous variables were analyzed using two-tailed *t* tests or Mann–Whitney *U* tests, while categorical variables were analyzed using the chi-squared test or Fisher's exact test.

For comparisons of categorical outcomes, generalized linear mixed models (GLMMs) with a logit link were used, adjusting for sex (a baseline variable with significant differences) and colonoscopy indication (the latter considered clinically meaningful), with healthcare center included as a random effect. Adjusted odds ratios (aORs) with 95%CIs were reported for comparisons between the two groups. For continuous outcomes, GLMMs with a Poisson distribution and a log link were applied, adjusting for the same covariates and random effects. Adjusted incidence rate ratios (aIRRs) with 95%CIs were calculated to compare the two groups.

Given the number of statistical tests conducted, Bonferroni correction was applied to account for multiple comparisons. A total of 11 statistical tests (one for the primary end point and 10 for secondary outcomes) were subject to Bonferroni correction, resulting in an adjusted significance level of 0.0045 (0.05/11). All *P* values for the primary and secondary outcomes were interpreted at this adjusted threshold.

Statistical analyses were conducted using SPSS (version 26.0, IBM, New York, USA) and R (version 4.3.3, R Foundation for Statistical Computing, Vienna, Austria).

## Results

**Fig. 4:**
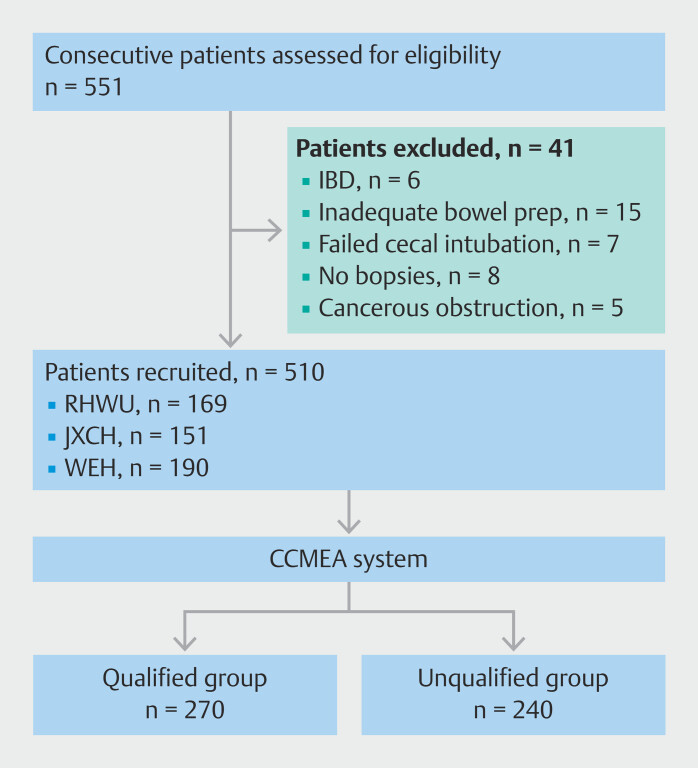
Flowchart of patient inclusion and exclusion in the prospective study. CCMEA, cumulative colorectal mucosal exposure area.

A total of 551 consecutive patients undergoing colonoscopy were screened between 1 October 2024 and 30 November 2024. There were 41 patients who were excluded before or during the procedure (suspected IBD [n = 6], inadequate bowel preparation [n = 15], failed cecal intubation [n = 7], no biopsies [n = 8], and cancerous obstruction [n = 5]). Finally, 510 eligible patients were included in the analysis (169 from RHWU, 151 from JXCH, and 190 from WEH) ( Fig. 4 ). Patient information was collected prospectively, without there being any missing values. The baseline characteristics of the patients (238 men [46.7%]; average age 58.5) are shown in **Table 1s** .

Based on a CCMEA of 2000 as the threshold, the patients were divided into the qualified group (n = 270) and the unqualified group (n = 240). On their baseline information, there were significant differences between the two groups in terms of sex and healthcare center ( Table 1 ). The detection rates of all types of colorectal lesions were higher in the qualified group than in the unqualified group. After adjusting for confounding factors, the detection rates of all types of lesions in the qualified group remained significantly different from those in the unqualified group ( Table 2 ).

**Table:** **Table 1** Comparison of the baseline information for the qualified and unqualified group in the prospective validation dataset.

Characteristics	Qualified group (n = 270)	Unqualified group (n = 240)	*P* value
Age, mean (SD), years	59.2 (10.6)	57.9 (12.0)	0.22
Sex, male, n (%)	146 (54.1)	92 (38.3)	<0.001
Body mass index, mean (SD), kg/m2	24.0 (3.4)	23.4 (3.9)	0.06
Indication for colonoscopy, n (%)			0.40
Health examination	62 (23.0)	49 (20.4)	
Diagnostic	160 (59.3)	156 (65.0)	
Surveillance	48 (17.8)	35 (14.6)	
Endoscope manufacturer, n (%)			0.45
Olympus	176 (65.2)	164 (68.3)	
Fujifilm	94 (34.8)	76 (31.7)	
Boston score, mean (SD)	7.89 (1.18)	7.94 (1.15)	0.64
Healthcare center, n (%)			<0.001
RHWU	106 (39.3)	63 (26.3)	
WEH	116 (43.0)	74 (30.8)	
JXCH	48 (17.8)	103 (42.9)	
RHWU, Renmin Hospital of Wuhan University; WEH, Wuhan Eighth Hospital; JXCH, JingXing County Hospital.			

**Table:** **Table 2** Comparison of the primary and secondary outcomes for the qualified and unqualified groups in the prospective validation dataset.

Outcome	Qualified group (n = 270)	Unqualified group (n = 240)	Absolute difference (95%CI)	Adjusted ratios ^1^ (95%CI)	*P* value ^1^
Detection rates			Percentage points	Adjusted OR	
Adenoma detection rate, n (%)2	145 (53.7)	34 (14.2)	39.5 (31.8–46.5)	7.8 (5.0–12.8)	<0.001
Adenoma size, n (%), mm ^2^					
Diminutive (≤5)	114 (42.2)	24 (10.0)	32.2 (25.0–39.0)	6.9 (4.1–11.5)	<0.001
Small (>5 to <10)	55 (20.4)	9 (3.8)	16.6 (11.2–22.1)	5.8 (2.7–12.3)	<0.001
Large (≥10)	14 (5.2)	1 (0.4)	4.8 (2.0–8.1)	12.2 (1.6–91.6)	0.02
Advanced adenoma rate, n (%) ^2^	16 (5.9)	2 (0.8)	5.1 (2.0–8.6)	7.7 (1.7–34.2)	0.007
Polyp detection rate, n (%) ^2^	242 (89.6)	96 (40.0)	49.6 (42.1–56.4)	13.1 (7.8–21.8)	<0.001
Polyp size, n (%), mm ^2^					
Diminutive (≤5)	222 (82.2)	83 (34.6)	47.6 (39.7–54.7)	10.0 (6.3–15.8)	<0.001
Small (>5 to <10)	77 (28.5)	21 (8.8)	19.8 (13.2–26.2)	4.5 (2.6–7.9)	<0.001
Large (≥10)	19 (7.0)	3 (1.3)	5.8 (2.4–9.6)	5.8 (1.7–20.2)	0.006
Detected numbers			Lesion numbers	Adjusted IRR	
Number of adenomas, mean (SD) ^3^	1.0 (1.3)	0.2 (0.5)	0.8 (0.7–1.0)	5.9 (4.2–8.4)	<0.001
Number of polyps, mean (SD) ^3^	5.8 (6.4)	1.3 (3.8)	4.5 (3.5–5.4)	4.0 (3.5–4.5)	<0.001
GLMM, generalized linear mixed model; IRR, incident rate ratio; OR, odds ratio. ^1^ Adjusted P values, adjusted ORs, and adjusted IRRs were derived from GLMMs. ^2^ Categorical variables were analyzed using GLMMs with a logit link, adjusting for sex and colonoscopy indication, with healthcare center as a random effect. P values <0.0045 (0.05/11) were considered significant as Bonferroni correction was adopted for multiple tests. ^3^ Continuous variables were analyzed using GLMMs with a Poisson distribution and a log link, adjusting for sex and colonoscopy indication, with healthcare center as a random effect. P values <0.0045 (0.05/11) were considered significant as Bonferroni correction was adopted for multiple tests.					

There was a 39.5 percentage point difference in ADR, with the qualified group showing a higher rate than the unqualified group (53.7% vs. 14.2%; aOR 8.0, 95%CI 5.0–12.8; *P* < 0.001). Similarly, the qualified group outperformed the unqualified group for adenoma detection across the various sizes, and particularly for lesions ≤5 mm (42.2% vs. 10.0%; aOR 6.9, 95%CI 4.1–11.5; *P* < 0.001). Furthermore, the detection rate of advanced adenomas in the qualified group was higher than that in the unqualified group (5.9% vs. 0.8%; aOR 7.7, 95%CI 1.7–34.2; *P* = 0.007).

In terms of polyp detection, the qualified group had a significantly higher PDR than the unqualified group (89.6% vs. 40.0%; aOR 13.1, 95%CI 7.8–21.8; *P <* 0.001). This difference was again particularly notable for lesions ≤5 mm (82.2% vs. 34.6%; aOR 10.0, 95%CI 6.3–15.8; *P <* 0.001). Additionally, the qualified group had significantly higher mean numbers of both adenomas (1.0 vs. 0.2; aIRR 5.9, 95%CI 4.3–8.4; *P <* 0.001) and polyps detected (5.8 vs. 1.3; aIRR 4.0, 95%CI 3.5–4.5; *P <* 0.001).

## Discussion

In this study, we proposed a novel indicator, the CCMEA, to comprehensively evaluate the quality of colonoscopy. Specifically, we employed deep learning models to develop an AI system capable of automatically calculating the CCMEA. The system assesses whether endoscopists have conducted the procedure adequately and comprehensively.

Missed diagnosis of colorectal neoplasia is the most common cause of PCCRC 11 12 ; 57.8% of PCCRC cases are attributed to neoplastic lesions missed during colonoscopy 13 . Therefore, the most important goal of colonoscopy is to maximize the detection of lesions, especially adenomas. Detection of lesions largely relies on the thorough examination of the entire mucosa by endoscopists; however, variations in endoscopists' technical proficiency can lead to significant differences in colonoscopy quality. Conventional colonoscopy quality indicators, such as the withdrawal time, lesion detection, and the Boston Bowel Preparation Scale (BBPS) score, rely on manual scoring and have various limitations.

To address these limitations, we proposed a new quality control indicator, the CCMEA, which can be calculated automatically. This automated, objective, and accurate assessment system of the colorectal mucosal exposure area helps evaluate whether endoscopists have performed the operation adequately and comprehensively. By setting the threshold for the CCMEA on the basis of the 25% ADR standard for colonoscopy, a threshold of 2000 was proposed for the CCMEA as an indicator of a qualified colonoscopy. We found that ADR was significantly higher in patients whose CCMEA was above the threshold. CCMEA was strongly correlated with ADR, and was better than the traditional colonoscopy quality index of standard withdrawal time. The CCMEA is expected to become a new intelligent-era quality control indicator for colonoscopy.

Compared with previous indicators, the CCMEA provides a more refined and practical evaluation of colonoscopy mucosal exposure. Liu et al. developed an AI-based system to assess the quality of fold exposure (FEQ) during colonoscopy, using the classification model to differentiate lumen and wall views, and calculating the visible folds proportion for the assessment of withdrawal performance during the withdrawal process 14 . This study showed that the system's evaluation of FEQ was strongly correlated with ADR and withdrawal time. Compared with the FEQ study, the CCMEA, as a new evaluation indicator, expanded the assessment scope of mucosal observation. By segmenting effective and ineffective mucosal exposure areas frame by frame, it directly quantified the proportion of mucosal exposure during colonoscopy. Compared with the classification model, the segmentation model can provide more refined mucosal exposure information and directly focuses on the visible area of the entire mucosa. The evaluation index is more intuitive, which is conducive to the understanding and application of endoscopists, especially novice endoscopists, and can reduce the rate of missed diagnosis of lesions and provide more accurate evaluation tools. By accumulating real-time the effective exposure area across frames, the CCMEA reflects full withdrawal-phase mucosal coverage and enables instant feedback, aligning better with the clinical needs of endoscopists, and may have greater practicality in operational guidance.

Poor bowel preparation, inadequate examination of mucosal folds, incomplete dilation of the colorectal cavity, and short withdrawal time can lead to blind areas during colonoscopy 15 16 . Among these, the withdrawal time has been identified as an important quality indicator of mucosal observation quality 17 , predicting the detection of adenomas 18 ; however, some studies have reported that the time of device withdrawal was not necessarily associated with a significant increase in lesion detection rate 19 , and that there is no relationship with the risk of PCCRC 20 . Therefore, withdrawal time remains a controversial indicator for quality control 21 . Calculation of the CCMEA is associated with many factors, such as withdrawal time, withdrawal speed, endoscopist's ability to stably observe the mucosa, and bowel preparation. Compared with withdrawal time, which is only associated with the withdrawal speed, the CCMEA can more comprehensively evaluate the quality of mucosal examination, partly representing the withdrawal time, bowel preparation, and mucosal exposure. Our study showed that the CCMEA was superior to standard withdrawal time in terms of detecting adenomas and polyps ( **Fig. 1s** ). Therefore, the CCMEA may be a better indicator of quality than the traditional standard withdrawal time.

During colonoscopy, our software can collect and analyze relevant data in a real-time manner using intelligent algorithms. A CCMEA below the qualified threshold is an alarm sign. The index and analysis results are presented to endoscopists through visual representation of colorectal mucosal area and dashboard indicators, accompanied by operational suggestions. Additionally, a professional quality control platform is used for feedback. In terms of hardware, the CPU is an Intel Core i7–9700K running at 3.60 GHz. The GPU is an NVIDIA 3070 with 8 GB of video random access memory (VRAM). In terms of software, we used the NVIDIA TensorRT framework. We adopted the half-precision floating-point (FP16) quantization technique to optimize the DCNN model. The above configuration is sufficient to construct a clinically applicable CCMEA system with real-time feedback capabilities.

Our study has certain limitations. First, CCMEA thresholds were retrospectively established using data from a single-center and applied to a multicenter prospective cohort, with all of the participating centers located only within China and endoscopic equipment limited to specific manufacturers (Olympus and Fujifilm). **Fig. 2s** shows the corresponding CCMEA vs. ADR curve, whose consistent trends across datasets support the CCMEA's potential as a quantifiable indicator for enhancing colonoscopy quality in diverse clinical scenarios. While this design strengthened generalizability within the included settings, there are differences in terms of diet, lifestyle, and ADR/PDR, not only across regions but also across countries, alongside potential variations in performance across diverse endoscopic systems globally. Notably, after Bonferroni correction, some secondary outcomes did not reach statistical significance, which is likely associated with the low rates of these outcomes, as such low detection rates may have reduced statistical power to detect potential associations. Moving forward, addressing these limitations will require large-scale international multicenter studies spanning diverse countries, ethnicities, healthcare settings, and endoscopic devices to confirm the CCMEA's robustness as a universal quality indicator.

Second, this study was an observational study, and further randomized controlled trials should be conducted to clarify the auxiliary effect of the CCMEA system on the colonoscopy operation of endoscopists, especially novice endoscopists.

Third, the applicability of the CCMEA in colonoscopies using mucosal exposure-enhancing devices needs careful consideration. These devices improve adenoma detection by enhancing mucosal visualization, which may alter the relationship between colonoscopy movement patterns and the ADR 22 . The Endocuff device, for example, increased ADR in randomized controlled trials 23 . Given this, the CCMEA, originally developed based on standard colonoscopy movement dynamics, may underestimate or overestimate procedural quality in device-assisted procedures, and adjustments to its calculation, such as accounting for device-specific visualization enhancements, might be necessary to maintain validity. Because the availability and routine use of these devices varies across different regions and healthcare settings, the present study did not include distal attachment device-assisted colonoscopy, which was necessary to determine the effect of the CCMEA in standard colonoscopy; however, this limitation underscores the need for device-specific validation of the CCMEA. Future studies should directly evaluate its performance in colonoscopies using mucosal exposure-enhancing devices, explore whether modifications to the metric (e.g. recalibrating movement–visibility correlations) are required, and develop targeted assessment methods tailored to such devices.

Fourth, this study excluded participants with a BBPS score <6, limiting the generalizability of our findings across populations with poor bowel preparation. Calculation of the CCMEA is highly dependent on the quality of endoscopic images. When bowel preparation is inadequate, in accordance with guidelines, repeat bowel preparation is needed for a qualified colonoscopy, rather than direct calculation of the CCMEA, which may be meaningless. While this exclusion was necessary to ensure the validity of the CCMEA in high quality examinations, it could introduce a selection bias and limit the applicability of the CCMEA to patients with adequate bowel preparation (BBPS ≥6). Therefore, it is necessary to conduct studies specifically focusing on the validity of the CCMEA in patients with insufficient bowel preparation. We are conducting a study to develop a precise evaluation system for assessing bowel preparation quality, especially for those with BBPS <6, which is expected to explore the application value of the CCMEA in colonoscopy with poor bowel preparation and BBPS <6, and expand its utility across diverse clinical scenarios in the future.

Finally, the CCMEA does not account for the mucosa hidden behind folds or requiring retroflexion. From the technical point of view, the classification model should be used to distinguish whether the fold is extended or not, and different coefficients should be used for the CCMEA; however, it is difficult to evaluate hidden mucosa, which is not visible to the naked eye.

In conclusion, we have proposed an AI-based CCMEA system for a more accurate assessment of the exposed area of colorectal mucosa. The system can evaluate the clear exposure area of the colorectal mucosa in a real-time manner during colonoscopy, which is expected to be an important tool for colonoscopy quality control.

## Supplementary Material

Supplementary Material Zusatzmaterial
