# Supplementary material for: Colorectal mucosal exposure area assessment using artificial intelligence: a multicenter prospective observational study
Source: Endoscopy. 2025 Oct 17;58(3):275–83. doi: 10.1055/a-2695-1832 (PMC13077554; doi:10.1055/a-2695-1832)
Supplement: Supplementary file 1 — Supplementary Material [file 10-1055-a-2695-1832_27155857.pdf]

Supplementary material

Colorectal mucosal exposure area assessment using artificial intelligence: a multicenter prospective observational study

Jialing Li*, Li Huang*, Chaijie Luo, Xiaoquan Zeng, Ying Li, Jianping Fan, Liwen Yao, Jing Wang, Shuzhe Tan, Xueying Wang, Wei Zhou, Lianlian Wu, Dexin Gong, Yuliang Xu, Muqiu Li, Ningning Wang, Huafen Gao, Honggang Yu
* Joint first authors

Appendix 1s Model development

Colonoscopy video would be processed into images set at 10 frames per second and the image set would be transferred to DCNN1. DCNN1 was employed to classify qualified and unqualified images. DCNN2 segmented the clearly exposed mucosa within the endoscopy view of qualified image, which were used as the molecules for calculation of CMEA. DCNN3 segmented the endoscopy view of qualified image within the frame, which was used as the denominator of CMEA calculation to eliminate the bias caused by the inconsistency of image regions between different endoscopic vendors or types. We employed a perceptual hashing algorithm (PHA) to identify and remove highly similar frames and eliminate redundant frames, thereby ensuring a more accurate CCMEA computation.[1,2]

In total, 3701 colonoscopy images in 2022 were exported from the Endoscopy Center of Renmin Hospital of Wuhan University (RHWU) and used as the image set for model construction and image labeling. In vitro, blurred, and flushing images, images with Boston bowel preparation scale (BBPS) < 2, and images of instruments, operations, or chromoendoscopy were excluded. For tranining DCNN2 and DCNN3, 3597 and 104 labeled images were employed respectively. The image dataset was randomly divided into the training set, validation set and test set with an 8:1:1 ratio.

Both DCNN2 and DCNN3 adopted UNet++ as their framework architecture, and all networks were trained on an NVIDIA GeForce GTX 2080 SUPER. Training and optimization were performed using Google's Keras 2.2.4, based on the TensorFlow 2.4.1 deep learning framework.[3] To minimize the risk of overfitting, three strategies were employed: dropout,[4] data augmentation,[5] and early stopping.[6] First, a dropout rate of 0.5 was applied to the input of the first and second fully connected layers in both models. Second, random image augmentations, including adjustments in height, width, and scaling, were used to for data argumentation. Through augmentation, images appeared differently in each training epoch. The augmentation techniques included: X randomly offset the image width from 0 to 0.05, and Y randomly offset the image height from 0 to 0.05; random rotation in the range of $-0.2*180^\circ$ to $0.2*180^\circ$; the range of tangent transformation angle was 0.05*180 degrees; random scaling by 0.95-1.05 times; random vertical flips and horizontal flips. Third, early stopping was applied by monitoring the validation curve, with training halted if the validation loss did not decrease over the latest 20 epochs. Binary cross-entropy was used as the loss function, and the Adam optimizer with a learning rate of 0.0001 was employed. Dice coefficient and mean intersection over union (mIoU) were adopted to evaluate the performance of segmentation models.

CCMEA vs. SWT in Predicting Adenoma and Polyp Detection

The area under the receiver operating characteristic curve (AUROC) was used to assess the association between CCMEA and ADR, determine the predictive value of CCMEA for ADR, and compare CCMEA and SWT across various outcomes. CCMEA demonstrated a significantly higher AUROC than SWT for detecting adenoma (0.73[95%CI 0.70-0.77] vs. 0.69[95%CI 0.65-0.74], p=0.001) (**Figure S1**), while the difference for polyp detection did not reach the threshold of statistical significance (0.76[95%CI 0.72-0.80] vs. 0.73[95%CI 0.69-0.78], p=0.132). Similarly, in the prospective validation dataset, CCMEA exhibited superior AUROC values compared to SWT for detecting adenoma (0.78[95%CI 0.74-0.82] vs. 0.72[95%CI 0.68-0.77], p<0.001) and polyp (0.86[95%CI 0.82-0.89] vs. 0.82[95%CI 0.79-0.86], p=0.008).

Table 1s Baseline information for threshold dataset and prospective validation dataset

Characteristics	Threshold dataset (n=716)	Prospective validation dataset (n=510)	*p* value
Age, mean(SD), years	58.73(8.31)	58.57 (11.28)	0.782
Sex, n(%)			0.435
Male	318(44.41)	238(46.67)	
Female	398(55.59)	272(53.33)	
Body mass index, mean(SD), kg/m^2	23.25(3.54）	23.65(3.63)	0.053
Indication for colonoscopy, n(%)			0.074
Health examination	119(16.62)	111(21.76)	
Diagnostic	476(66.48)	316(61.96)	
Surveillance	121(12.9)	83(16.27)	
Endoscope vendor, n(%)			0.747
Olympus	471(65.78)	340(66.67)	
Fujifilm	245(34.22)	170(33.33)	
Boston score, mean(SD)	7.97(0.91)	7.91(1.16)	0.328

SD, standard deviation.

Figure 1s AUROC of CCMEA versus SWT in terms of presence of adenoma and polyp

(A) AUROC for the presence of adenoma in threshold dataset. (B) AUROC for the presence of polyp in threshold dataset. (C) AUROC for the presence of adenoma in prospective validation dataset. (D) AUROC for the presence of polyp in prospective validation dataset.

CCMEA, Cumulative colorectal mucosal exposure area; *SWT*, standard withdrawal time; *AUROC*, area under receiver operating characteristic curve.

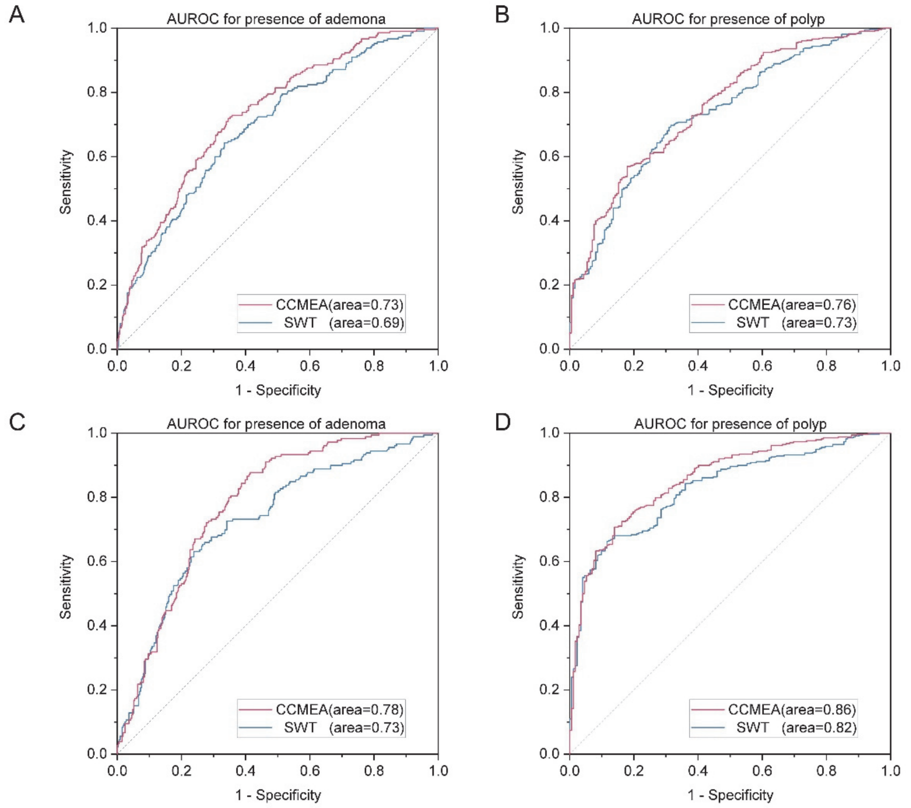

Figure 2s Validation of CCMEA Threshold in the Prospective Dataset

Consistent with the grouping strategy by CCMEA per 1000, the prospective dataset validated that a CCMEA of 2000 remained the threshold associated with an ADR exceeding 25%.

CCMEA, Cumulative colorectal mucosal exposure area; *ADR*, Adenoma detection rate.

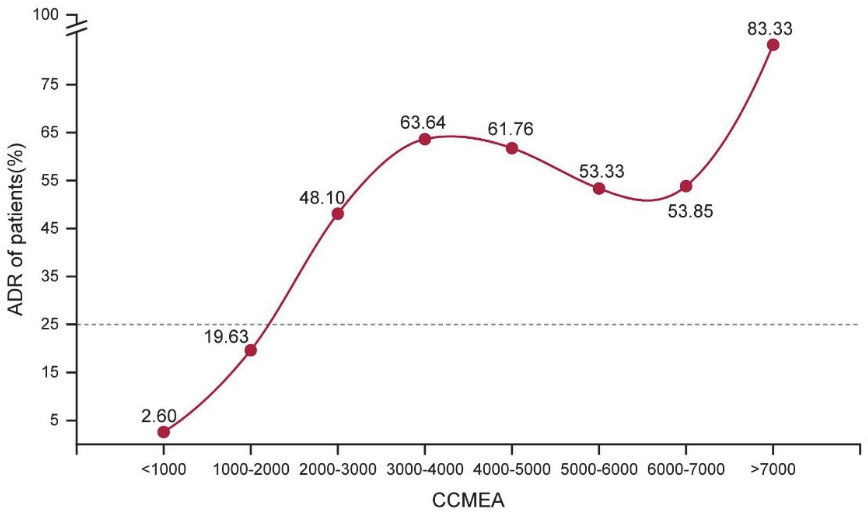

Supplementary references

1 Gong D, Wu L, Zhang J, *et al*. Detection of colorectal adenomas with a real-time computer-aided system (ENDOANGEL): a randomised controlled study. *Lancet Gastroenterol Hepatol* 2020;**5**(4):352-361.

2 Yao L, Zhang L, Liu J, et al Effect of an artificial intelligence-based quality improvement system on efficacy of a computer-aided detection system in colonoscopy: A four-group parallel study. *Endoscopy* 2022;**54**(08): 757–68.

3 Abadi M, Barham P, Chen J, *et al*, editors. Tensorflow: A system for large-scale machine learning. 12th {USENIX} Symposium on Operating Systems Design and Implementation ({OSDI} 16); 2016.

4 Baldi P, Sadowski P. The dropout learning algorithm. *Artificial intelligence* 2014;**210**:78-122.

5 Mikołajczyk A, Grochowski M, editors. Data augmentation for improving deep learning in image classification problem. 2018 international interdisciplinary PhD workshop (IIPhDW); 2018: IEEE.

6 Duvenaud D, Maclaurin D, Adams R, editors. Early stopping as nonparametric variational 48 inference. Artificial Intelligence and Statistics; 2016.
